# N-cadherin in cancer metastasis, its emerging role in haematological malignancies and potential as a therapeutic target in cancer

**DOI:** 10.1186/s12885-018-4845-0

**Published:** 2018-10-01

**Authors:** Krzysztof Marek Mrozik, Orest William Blaschuk, Chee Man Cheong, Andrew Christopher William Zannettino, Kate Vandyke

**Affiliations:** 1 Myeloma Research Laboratory, Adelaide Medical School, Faculty of Health and Medical Sciences, The University of Adelaide, Adelaide, Australia; 2 Cancer Theme, South Australian Health and Medical Research Institute, Adelaide, Australia; 3 Division of Urology, Department of Surgery, McGill University, Montreal, Canada; 4 Centre for Cancer Biology, University of South Australia, Adelaide, Australia

**Keywords:** N-cadherin, Cancer, Metastasis, Haematological malignancies, Therapeutic target

## Abstract

In many types of solid tumours, the aberrant expression of the cell adhesion molecule N-cadherin is a hallmark of epithelial-to-mesenchymal transition, resulting in the acquisition of an aggressive tumour phenotype. This transition endows tumour cells with the capacity to escape from the confines of the primary tumour and metastasise to secondary sites. In this review, we will discuss how N-cadherin actively promotes the metastatic behaviour of tumour cells, including its involvement in critical signalling pathways which mediate these events. In addition, we will explore the emerging role of N-cadherin in haematological malignancies, including bone marrow homing and microenvironmental protection to anti-cancer agents. Finally, we will discuss the evidence that N-cadherin may be a viable therapeutic target to inhibit cancer metastasis and increase tumour cell sensitivity to existing anti-cancer therapies.

## Background

Cancer metastasis is a leading cause of cancer-related mortality. The metastasis of cancer cells within primary tumours is characterised by localised invasion into the surrounding microenvironment, entry into the vasculature and subsequent spread to permissive distant organs [1, 2]. In many epithelial cancers, metastasis is facilitated by the genetic reprogramming and transitioning of cancer cells from a non-motile, epithelial phenotype into a migratory, mesenchymal-like phenotype, a process known as epithelial-to-mesenchymal transition (EMT) [3, 4]. A common feature of EMT is the loss of epithelial cadherin (E-cadherin) expression and the concomitant up-regulation or de novo expression of neural cadherin (N-cadherin). This so-called “cadherin switch” is associated with increased migratory and invasive behaviour [5, 6] and inferior patient prognosis [7–10]. A major consequence of E-cadherin down-regulation is the loss of stable epithelial cell-cell adhesive junctions, apico-basal cell polarity and epithelial tissue structure, thereby facilitating the release of cancer cells from the primary tumour site [11, 12]. In contrast to the migration-suppressive role of E-cadherin, N-cadherin endows tumour cells with enhanced migratory and invasive capacity, irrespective of E-cadherin expression [13]. Thus, the acquisition of N-cadherin appears to be a critical step in epithelial cancer metastasis and disease progression.

In this review, we will discuss how N-cadherin promotes the metastatic behaviour of tumour cells by directly mediating cell-cell adhesion, and by its involvement in modulating critical signalling pathways implicated in metastatic events. In addition, we will discuss the emerging relevance of N-cadherin in haematological malignancies, namely leukaemias and multiple myeloma. Finally, we will review the emerging evidence that N-cadherin may be a viable therapeutic target to inhibit cancer metastasis and overcome resistance to anti-cancer agents.

## Structure and formation of the N-cadherin adhesive complex

**Fig. 1:**
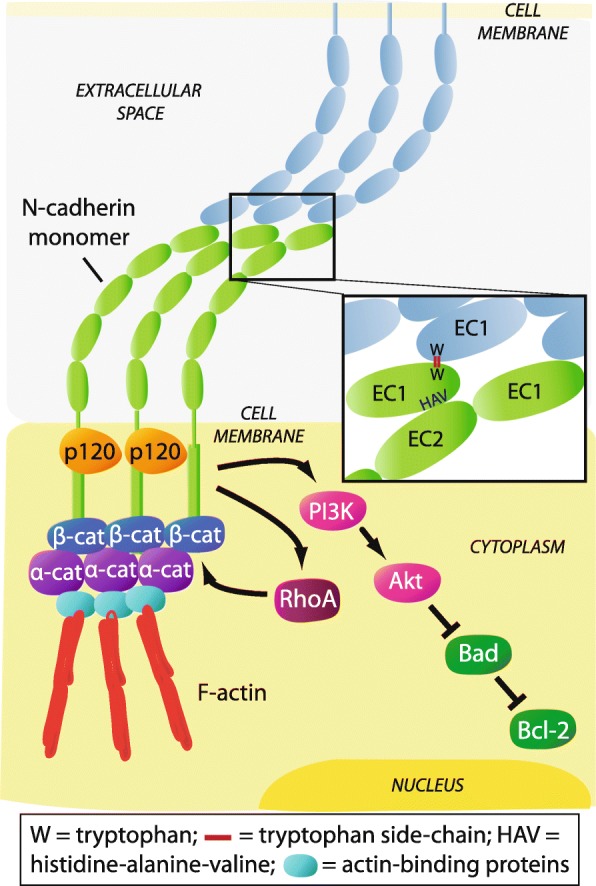
Schematic representation of the N-cadherin-catenin adhesive complex. The extracellular domains of N-cadherin monomers engage in *trans* and *cis* interactions with partner monomers, facilitated by p120-catenin (p120), resulting in a lattice-like arrangement. Interaction between monomers on opposing cells occurs via a reciprocal insertion of tryptophan side-chains (W) on the first extracellular domain (EC1) (*trans* adhesion). Clustering of N-cadherin monomers on the same cell occurs via a His-Ala-Val (HAV) adhesion motif on EC1 and a recognition sequence on the second extracellular domain (EC2) of the partner monomer (*cis* adhesion) (inset). Activation of RhoA sequesters β-catenin (β-cat) and results in accumulation of α-catenin (α-cat) to the N-cadherin intracellular domain. This promotes anchorage of the N-cadherin-catenin complex to the actin cytoskeleton via actin-binding proteins, thereby stabilising cell-cell contacts. Initial ligation of N-cadherin extracellular domains also triggers PI3K/Akt signalling which inactivates the pro-apoptotic protein Bad, resulting in activation of the anti-apoptotic protein Bcl-2

N-cadherin is a member of the calcium-dependent adhesion molecule family of classical cadherins which directly mediate homotypic and heterotypic cell-cell adhesion. N-cadherin is a classical type I cadherin consisting of 5 extracellular domains linked to a functional intracellular domain. The engagement between N-cadherin monomers on opposing cells occurs by reciprocal insertion of a tryptophan residue side-chain on its first extracellular domain (EC1) into the hydrophobic pocket of the partner N-cadherin EC1 (*trans* adhesion). In addition, the stabilisation of N-cadherin-mediated adhesion requires the clustering of adjacent monomers on the surface of the same cell, involving the His-Ala-Val (HAV) motif on EC1 and a recognition sequence on the second extracellular domain (EC2) of the lateral N-cadherin monomer (*cis* adhesion) [14–16]. The membrane expression and lateral clustering of N-cadherin is dependent upon p120 catenin, which localises N-cadherin at cholesterol-rich microdomains [17, 18]. The initial ligation of N-cadherin extracellular domains triggers the activation of the Rho GTPase family member Rac, which stimulates localised actin filament assembly and the formation of membrane protrusions at points of cell-cell contact [19, 20]. The subsequent activation of the Rho GTPase family member RhoA, at the expense of Rac function, facilitates the maturation of N-cadherin-based cell-cell junctions by triggering the sequestration of β-catenin to the cadherin intracellular domain [21, 22]. β-catenin serves as a critical link to α-catenin which accumulates at nascent cell-cell junctions and suppresses actin branching. In addition, α-catenin facilitates the anchorage of the N-cadherin-catenin complex to the actin cytoskeleton via actin-binding proteins such as cortactin and α-actinin, thereby promoting the maturation of cell-cell contacts [23, 24] (Fig. 1). Notably, the adhesive function of N-cadherin is regulated by post-translational modifications of the N-cadherin-catenin complex. For instance, the stability of the N-cadherin-catenin complex is highly dependent on the phosphorylation status of N-cadherin and the associated catenins, which is regulated by tyrosine kinases, such as Fer and Src, and the tyrosine phosphatase PTP1B [25, 26]. In addition, branched *N*-glycosylation of N-cadherin EC2 and third extracellular domain regulates N-cadherin-dependent cell adhesion, at least in part, by controlling the lateral clustering of N-cadherin monomers [27].

## The functional role of N-cadherin in solid tumour metastasis

N-cadherin expression is spatiotemporally regulated throughout development and adulthood. In development, N-cadherin plays an important role in morphogenetic processes during the formation of cardiac and neural tissues, and is involved in osteogenesis, skeletal myogenesis and maturation of the vasculature [28–32]. In adulthood, N-cadherin is expressed by numerous cell types including neural cells, endothelial cells, stromal cells and osteoblasts, and is integral to synapse function, vascular stability and bone homeostasis [30, 33–36]. While N-cadherin is typically absent or expressed at low levels in normal epithelial cells, the aberrant expression of N-cadherin in epithelial cancer cells is a well-documented feature of epithelial malignancies, such as breast, prostate, urothelial and pancreatic cancer, and is associated with disease progression [37–40]. In a similar manner, the up-regulation of N-cadherin expression is a feature of melanoma progression [41–43]. Whilst the aberrant expression of N-cadherin in epithelial tissues is not considered to be oncogenic, or a promoter of solid tumour growth [44–46], increased expression of N-cadherin in cancer is widely associated with tumour aggressiveness. Indeed, many studies have demonstrated a significant correlation between elevated N-cadherin levels in epithelial, and some non-epithelial solid tumours, and clinicopathologic features such as increased localised tumour invasion and distant metastasis, and inferior patient prognosis [7, 8, 47–81] (Table 1). Multivariate analyses have also identified that elevated N-cadherin expression is independently associated with inferior patient prognosis in several epithelial malignancies including prostate, lung and bladder cancer [8, 55, 56, 60, 62, 63, 67, 72, 78, 80] (Table 1). The aggressive phenotype and inferior prognosis associated with up-regulated N-cadherin expression in solid tumours is also supported by a recent meta-analysis incorporating patients with various epithelial malignancies [82].

**Table 1:** Association of increased N-cadherin expression in cancer with clinicopathologic features and survival

Cancer type	Cohort information & treatment details	No. of patients	N-cadherin detection method	Association with clinicopathologic features	Association with survival	Reference
Epithelial cancers						
Breast cancer	Pre-metastatic; resected	574	IHC	High grade & LN metastasis	Shorter PFS (U)	[47]
	Early-stage invasive	1902	IHC	Earlier development of distant metastasis	n/a	[48]
	Primary inoperable and LN negative	275	IHC	n.s.	Shorter OS (U)	[49]
	Invasive; no prior therapy	94	IHC	High grade, late stage & LN metastasis	n/a	[50]
Prostate cancer	Clinically localised; radical prostatectomy	104	IHC	Poor differentiation, seminal vesicle invasion & pelvic LN metastasis	Shorter time to biochemical failure (U), clinical recurrence (M) & skeletal metastasis (U)	[8]
	Castration-resistant; transurethral resection	26	IHC	Higher Gleason score & metastasis	n/a	[51]
	Localised; no therapy prior to radical prostatectomy	157	IHC	Later stage, higher PSA & Gleason score, seminal vesicle invasion and LN metastasis	n/a	[52]
	Blood from cancer follow-up patients	179	Serum ELISA (sN-cad)	Higher PSA	n/a	[53]
	Radical prostatectomy, metformin-treated	49	IHC	n/a	Increased recurrence	[54]
Lung cancer	Adenocarcinoma & squamous cell carcinoma; no therapy prior to surgery	68	IHC	Higher TNM stage & poor differentiation	Shorter OS (M)	[55]
	Primary adenocarcinoma; no therapy prior to surgery	147	IHC	n/a	Shorter OS (M)	[56]
	Surgical resection of adenocarcinoma; no prior therapy	57	qPCR	LN metastasis	n/a	[57]
	No post-operative surgery	186	IHC	Higher TNM stage & metastasis	n/a	[58]
	Adenocarcinoma & squamous cell carcinoma; blood collected prior to or up to 3 weeks after platinum-based therapy	43	IF (on CTCs)	n/a	Shorter PFS	[59]
Urothelial cancers	Radical cystecomy with pelvic LN dissection, clinically nonmetastatic bladder cancer	433	IHC	Higher clinical & pathologic tumour stage, LN metastasis & LN stage, lymphovascular invasion	Shorter RFS (M), OS (U) & cancer-specific survival (U)	[60]
	Invasive bladder cancer undergoing radical cystectomy; no prior treatment	30	qPCR	n/a	Shorter OS	[61]
	Transurethral resection of non-muscle-invasive bladder cancer	115	IHC	Higher incidence of intravesical recurrence	Shorter intravesical RFS (M)	[62]
	Clinically-localised upper urinary tract carcinoma undergoing nephroureterectomy; cisplatin- based therapy in late-stage patients	59	IHC	n/a	Intravesical and extravesical RFS (M)	[63]
Liver cancer	Resection of hepatocellular carcinoma	100	IHC	Higher histologic grade, multifocal tumours & vascular invasion	Shorter disease-free and OS	[64]
	Surgical resection of hepatocellular carcinoma	57	IHC	n.s.	Increased recurrence- rate within 2 years of resection	[65]
	Surgical resection of intrahepatic cholangiocarcinoma (no prior therapy); adjuvant therapy in patients with recurrence	96	IHC	Higher recurrence of vascular invasion	Shorter OS	[66]
Head & neck cancer	Surgical specimen of HNSCC, patients are +/− LN metastasis	119	IHC	Greater tumour size, higher clinical stage & LN metastasis	Shorter OS (M)	[67]
	Laryngeal, oripharyngeal & oral cancer; blood collected following HNSCC resection	10	IF	n/a	Shorter OS	[68]
	Radical surgery for laryngeal cancer; adjuvant therapy in 60% of cases	50	(on CTCs) IHC	Higher grade	Increased relapse	[69]
	Nasopharyngeal cancer	122	IHC	LN involvement, distant metastasis & later clinical stage	Shorter OS (nuclear N-cadherin)	[70]
Gastrointestinal tract cancer	Colorectal cancer; no therapy prior to surgery	37	qPCR	Local invasion, Dukes staging & vascular invasion	n/a	[71]
	Colorectal cancer; no therapy prior to surgery	102	IHC	Larger tumour size, poor differentiation, tumour invasion, LN metastasis & distant metastasis	Shorter OS (M) & shorter disease-free survival	[72]
	Colon carcinoma; no therapy prior to surgery	90	IHC	Greater depth of tumour invasion & higher TNM stage	n/a	[73]
	Gastric cancer surgery with LN metastasis; no prior therapy	89	IHC (on LN)	LN involvement, higher pathological stage, lymphatic invasion & venous invasion	Shorter OS	[74]
	Curative surgery for gastric adenocarcinoma; no prior therapy, stage II patients received adjuvant therapy	146	IHC	Haematogenous recurrence	Shorter survival	[75]
Renal cancer	Blood collected from metastatic renal cell carcinoma patients with prior nephrectomy and therapy	14	IF (on CTCs; also CK-)	n/a	Shorter PFS	[76]
Ovarian cancer	Surgical specimens of high-grade serous carcinoma	167	IHC	n/a	Shorter PFS and OS (U)	[77]
Gallbladder cancer	Adenocarcinoma (+/− surgery)	80	IHC	Poor differentiation, larger tumour size, TNM stage, invasion & LN metastasis	Shorter OS (M)	[78]
	Squamous cell/adenosquamous carcinoma (+/− surgery)	46	IHC	Larger tumour size, invasion and LN metastasis	Shorter OS (M)	[78]
Non-epithelial solid cancers						
Melanoma	Removal of primary melanoma, various stages of disease	394	IHC	Increased Breslow thickness	Distant metastasis-free survival (M; *p* = 0.13)	[7]
Sarcoma	Surgical resection of osteosarcoma	107	qPCR	Later stage and distant metastasis	Shorter survival	[79]
	Blood collected from a variety of bone & soft tissue sarcoma patients	73	Serum ELISA (sN-cad)	Larger tumour size & higher grade	Shorter disease-free survival (M) & OS (U)	[80]
Haematological malignancies						
Multiple myeloma	Blood collected from newly- diagnosed patients; no prior therapy	84	Serum ELISA (sN-cad)	n/a	Shorter PFS and OS	[81]
	Bone marrow aspirate from newly-diagnosed patients; no prior therapy	14	qPCR (on CD38+/CD138+ tumour cells)	n/a	Shorter PFS	[81]

All clinicopathologic and survival data shown is positively associated with increased N-cadherin expression. All data is statistically significant (*P* < 0.05), unless otherwise indicated. Abbreviations: *PFS* Progression-free survival, *RFS* Recurrence-free survival, *OS* Overall survival, *U* Univariate analysis, *M* Multivariate analysis, *IHC* Immunohistochemistry, *qPCR* Quantitative PCR, *IF* Immunofluorescence, *ELISA* Enzyme-linked immunosorbent assay, *sN-cad* Soluble N-cadherin, *PSA* Prostate specific antigen, *LN* Lymph node, *TNM* Tumour, node and metastases, *CTCs* Circulating tumour cells, *CK* Cytokeratin, *n/a* Not applicable, *n.s.* Not significant

Beyond the prognostic implications of aberrant N-cadherin expression, the relationship between N-cadherin and metastasis is not merely associative. Indeed, there is a wealth of evidence that increased N-cadherin expression enhances the migratory and invasive capacity of multiple epithelial cancer cell types in vitro [83–87]. The ability of N-cadherin to promote epithelial tumour metastasis in vivo was initially demonstrated using the MCF-7 breast cancer cell line, following injection into the mammary fat pad of nude mice. In contrast to wild-type cells, MCF-7 cells ectopically expressing N-cadherin formed tumour metastases in several organs including the liver, pancreas and lymph nodes [88]. Similarly, N-cadherin expression in the mammary epithelium in the transgenic MMTV-PyMT murine breast cancer model resulted in a three-fold increase in the number of pulmonary metastatic foci without affecting the onset or growth of the primary tumour [45]. Using an orthotopic mouse model of pancreatic cancer, the over-expression of N-cadherin in BxPC-3 cells increased the formation of disseminated tumour nodules throughout the abdominal cavity and induced the formation of N-cadherin-expressing lung micro-metastases [85]. Consistent with these findings, enforced expression of N-cadherin in androgen-responsive prostate cancer cells promoted invasion of underlying muscle and lymph node metastasis following subcutaneous injection in castrated mice [89]. Notably, N-cadherin also potentiates the invasiveness of melanoma cells. To this end, studies have demonstrated that N-cadherin promotes the capacity of melanoma cells to migrate on monolayers of dermal fibroblasts and undergo trans-endothelial migration in vitro [86, 90, 91]. Moreover, N-cadherin silencing has been shown to attenuate the ability of intravenously injected melanoma cells to extravasate and form lung metastases in immunocompromised mice [92].

To appreciate how N-cadherin, a cell adhesion molecule, may actively promote cancer cell migration, it is important to consider that the N-cadherin-catenin complex mediates both cell-cell adhesion and pro-metastatic cell signalling. Moreover, the adhesive function and migration-related signalling capacity of N-cadherin can occur simultaneously, or as antagonistic events, adding further complexity to its role in cancer metastasis. In the following section, we describe three key mechanisms by which N-cadherin has been shown to actively promote the migratory capacity of tumour cells: facilitation of collective cell migration, augmentation of fibroblast growth factor-receptor (FGFR) signalling and modulation of canonical Wnt signalling.

### N-cadherin promotes collective cell migration

The migration of cells as sheets, clusters or strands, a process termed collective cell migration, frequently occurs throughout development and in adulthood. For instance, collective cell migration occurs in embryogenesis, during gastrulation and neural crest cell migration, and in adult tissues, during wound healing and angiogenesis [93, 94]. In addition, collective cell migration facilitates the invasion of epithelial cells through the localised tumour host microenvironment, thereby promoting metastasis [95]. During this process, collectively migrating cells maintain physical interconnectivity, collective cell polarity and co-ordinated cytoskeletal activity, resulting in a ‘leader-follower’-type cellular arrangement. This promotes more efficient directional migration, in response to a chemotactic gradient, than that of an individual migrating cell [93, 96]. Adhesive complexes are integral to the co-ordinated behaviour of collectively migrating cells by mediating adhesion, signal transduction and mechanotransduction between adjacent cells [94, 97]. Notably, studies have demonstrated that N-cadherin expression by epithelial cancer cells promotes their capacity for collective migration. For instance, N-cadherin has been shown to promote the ability of lung or ovarian cancer cells to form aggregates and collectively invade three-dimensional (3D) collagen matrices or penetrate peritoneal mesothelium-like cell layers in vitro [87, 98]. Similarly, studies in transformed canine kidney epithelial cells (MDCK cells) have shown that N-cadherin promotes aggregate formation which allows directional collective cell migration in a 3D collagen matrix. In these cells, deletion of the entire N-cadherin intracellular domain, or the β-catenin binding domain alone, resulted in greater individual cell detachment and migration from cell clusters, highlighting the importance of the N-cadherin-actin cytoskeleton interaction in collective cell migration. Moreover, over-expression of an N-cadherin mutant in which the extracellular domain was fused to the anti-binding domain of α-catenin hindered the movement of follower cells, demonstrating that dynamic N-cadherin-actin linkage is required for efficient collective cell migration [99].

**Fig. 2:**
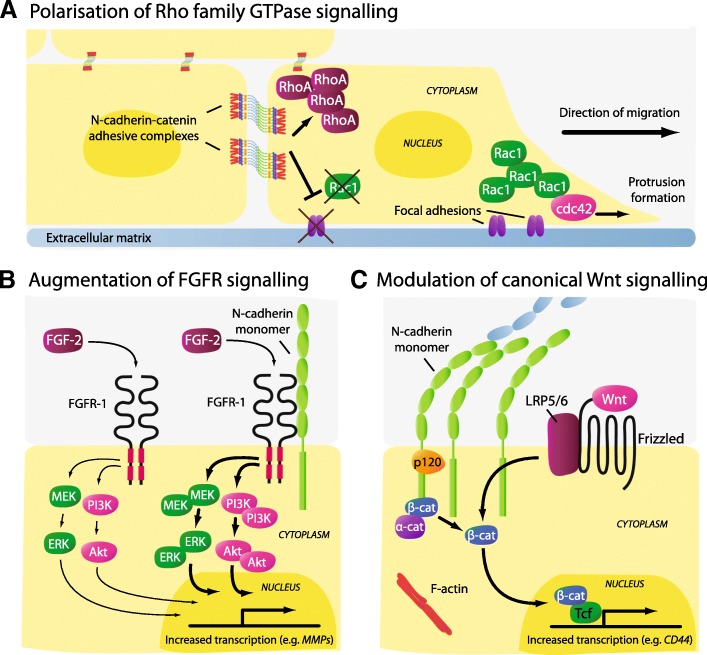
Schematic representation of cell signalling events modulated by increased N-cadherin expression in the context of cell migration. **a** In addition to mediating cellular aggregation, N-cadherin may facilitate the collective migration of tumour cells by excluding focal adhesions and Rac1 activity, and promoting RhoA activity, at sites of N-cadherin-mediated cell-cell contact. The asymmetric distribution of N-cadherin adhesive complexes polarises integrin function and Rac1 activity towards the free edges of cells, thereby directing focal adhesion and lamellipodia formation away from the cell cluster and promoting cell migration. Similar to Rac1, N-cadherin-mediated cell-cell adhesion promotes cdc42 activity at the free edges of cells, resulting in filipodia formation. **b** Functional interaction between the extracellular domains of N-cadherin and FGFR-1 potentiates FGF-2-activated FGFR-1 signalling by attenuating ligand-induced receptor internalisation. The resulting augmentation of down-stream MEK/ERK and PI3K/Akt signalling promotes the metastatic behaviour of cancer cells by increasing the production of invasion-facilitating molecules such as matrix metalloproteinases (MMPs). **c** N-cadherin-mediated adhesive complexes and Wnt/β-catenin signalling are thought to compete for the same cellular pool of β-catenin. While N-cadherin sequesters β-catenin from the nucleus, the N-cadherin adhesive complex provides a reservoir of β-catenin which, upon Wnt activation, becomes available for nuclear translocation and TCF/LEF-mediated gene transcription (e.g. *CD44* and MMP genes), resulting in the loss of N-cadherin-mediated cellular adhesion in cancer cells

In addition to maintaining multi-cellular aggregates of tumour cells, studies in N-cadherin-expressing non-tumour cells have demonstrated that N-cadherin also promotes collective cell migration by polarising Rho-family GTPase signalling (e.g. Rac1 and cdc42), known to co-ordinate cytoskeletal remodelling in collectively migrating cells [100, 101]. For example, models of arterial smooth muscle wound-healing and neural crest migration have shown that the asymmetric distribution of N-cadherin-mediated cell-cell adhesion at the lateral and posterior aspects of leader cells promotes directional cell alignment and increased cdc42 and Rac1 activity and protrusion formation at the free leading cell edge, resulting in enhanced migration [102, 103]. Mechanistically, studies in mouse embryonic fibroblasts have demonstrated that N-cadherin-adhesive complexes at the rear of cells suppress localised integrin-α5 activity, thereby polarising integrin and Rac activity towards the free leading edge of the cell [104]. Indeed, functional inhibition of N-cadherin in transformed mammary cells has been shown to reduce integrin-α5-dependent cell migration on fibronectin in vitro [105]. In a similar manner, silencing of N-cadherin expression in melanoma cells perturbs α2β1-integrin-dependent collagen matrix invasion in vitro [106]. Reciprocally, integrin signalling at focal adhesions has been shown to regulate the ability of HeLa cells to engage in N-cadherin-based connections and to promote collective cell migration [107]. Given that integrins play an important role in the activation of Rho signalling [108, 109], it is plausible that N-cadherin may polarise Rho-family GTPase signalling via intercommunication with integrins, thereby promoting the collective migration of cancer cells (Fig. 2a).

### N-cadherin augments fibroblast growth factor receptor signalling

Functional interaction between the extracellular domains of N-cadherin and receptor-tyrosine kinase FGFRs was first recognised as a mechanism by which N-cadherin promoted axonal outgrowth of rat cerebellar neuronal cells. These studies identified that the fourth extracellular domain of N-cadherin (EC4) trans-activated FGFRs to promote neurite outgrowth independent of FGF ligands, suggesting that N-cadherin can act as a surrogate ligand of FGFRs [33, 110]. The physical interaction of N-cadherin and FGFRs has also been shown in breast and pancreatic cancer cells [111–114]. Evidence that FGFR plays a functional role in N-cadherin-mediated cancer metastasis has been demonstrated in BT-20 and PyMT breast cancer cells, whereby FGFR inhibition reduced the in vitro migratory capacity of N-cadherin-expressing cells, but not N-cadherin-negative cells [45, 84]. In addition, FGF-2 increased the invasiveness of N-cadherin-expressing MCF-7 human breast cancer cells, but not control MCF-7 cells [88]. To this end, it has been shown that N-cadherin potentiates FGF-2-activated FGFR-1 signalling by attenuating ligand-induced FGFR-1 internalisation, thereby stabilising FGFR-1 expression [111, 113]. In turn, the sustained activation of down-stream MEK/ERK signalling results in increased production of the extracellular matrix (ECM)-degrading enzyme matrix metalloproteinase-9 (MMP-9) and enhanced breast cancer cell invasiveness [88, 111]. In addition, the interaction of N-cadherin and FGFR is also likely to promote metastasis by activation of the phosphatidylinositide-3 kinase/Akt (PI3K/Akt) signalling pathway in some cancer cell types. For example, studies suggest that the invasiveness of N-cadherin-expressing ErbB2/Neu breast cancer cells following FGFR activation is mediated by PI3K/Akt signalling. N-cadherin potentiates FGFR-Akt signalling and sensitivity to FGFR inhibition in ErbB2/Neu cells, suggesting the involvement of an N-cadherin-FGFR-PI3K/Akt signalling axis in breast cancer cell invasion [115] (Fig. 2b).

Two lines of evidence suggest that N-cadherin-FGFR-1 interactions promote the invasive behaviour in both collectively migrating and individual cancer cells. Firstly, N-cadherin-FGFR-1 interactions have been shown to occur over most of the cell membrane, but are excluded from sites of cell-cell adhesion, suggesting that the interaction is independent of N-cadherin-mediated cellular adhesion [112]. Secondly, blocking antibodies directed at the FGFR-1-interacting domain of N-cadherin (EC4) have been shown to inhibit N-cadherin-mediated migration, but not N-cadherin-mediated aggregation, of human breast cancer cells [116]. Thus, it would appear that N-cadherin-mediated cell-cell adhesion and N-cadherin-mediated cell migration via FGFR-1 are independent and mutually exclusive events. Further studies are warranted to identify whether N-cadherin potentiates FGFR-1 signalling in other epithelial malignancies such as pancreatic cancer.

### N-cadherin modulates canonical Wnt signalling

In addition to stabilising cadherin-mediated cell-cell adhesion, β-catenin plays a central role in the canonical Wnt signalling pathway. Canonical Wnt signalling promotes the cytoplasmic accumulation and nuclear translocation of β-catenin, which activates T cell factor/lymphoid enhancer factor (TCF/LEF)-mediated transcription of genes [117–119] that encode tumour invasion and metastasis-promoting molecules (e.g. MMPs and CD44) [120–126]. It has been proposed that cadherins and the canonical Wnt signalling pathway may compete for the same cellular pool of β-catenin, with cadherins sequestering β-catenin from the nucleus, thereby attenuating Wnt signalling [127, 128]. Indeed, enforced expression of N-cadherin in colon carcinoma cells resulted in the relocation of nuclear β-catenin to the plasma membrane and attenuated LEF-responsive trans-activation [129]. Alternatively, studies suggest that the N-cadherin-β-catenin complex may provide a stable pool of β-catenin available for TCF/LEF-mediated gene transcription in cancer cells [91, 130]. To this end, disruption of N-cadherin-mediated adhesion in leukaemic cells was found to increase TCF/LEF reporter activity [131]. Thus, given β-catenin is essential in the stabilisation of N-cadherin-mediated cellular adhesion (discussed earlier), it is feasible that the ability of N-cadherin to modulate TCF/LEF-mediated gene transcription may play an important role in individual cell migration, at the expense of collective cell migration (Fig. 2c).

Trans-endothelial migration is an important process in the haematogenous dissemination of cancer cells to distant sites [132]. Notably, studies suggest that N-cadherin promotes the trans-endothelial migration of cancer cells. To this end, N-cadherin silencing has been shown to reduce the ability of melanoma cells to undergo trans-endothelial migration in vitro [91]. Studies have demonstrated that N-cadherin-mediated melanoma cell adhesion to endothelial cells promotes trans-endothelial migration by modulating canonical Wnt signalling. β-catenin co-localises with N-cadherin during the initial stages of melanoma cell adhesion to endothelial cells; however, during transendothelial migration, the tyrosine kinase Src is activated and subsequently phosphorylates the N-cadherin cytoplasmic domain, thereby dissociating the N-cadherin-β-catenin complex. β-catenin is then translocated to the nucleus of melanoma cells and activates TCF/LEF-mediated gene transcription, resulting in up-regulation of the adhesion molecule CD44 [91, 133]. Studies using epithelial cancer cells suggest that CD44 binding to E-selectin on endothelial cells activates intracellular signalling pathways that lead to disassembly of endothelial junctions, thereby facilitating trans-endothelial migration [134–136]. In line with these studies, CD44 expression in melanoma cells has been shown to promote endothelial gap formation and trans-endothelial migration in vitro [137]. Moreover, N-cadherin knock-down in human melanoma cells reduces extravasation and lung nodule formation following intravenous injection in immuno-compromised mice [92]. Notably, while N-cadherin-expressing tumour cells have been detected in the circulation of patients with various epithelial cancers [59, 68, 76], and CD44 has been shown to promote diapedesis in breast cancer cells [134, 138], a role for N-cadherin in the trans-endothelial migration of epithelial cancer cells has not been directly demonstrated to date.

## The emerging role of N-cadherin in haematological malignancies

We have thus far summarised the functional role and clinical implications of aberrant N-cadherin expression in the context of solid tumour metastasis. There is now emerging evidence suggesting that N-cadherin plays a role in haematological malignancies, including leukaemia and multiple myeloma (MM). These cancers account for approximately 10% of all cancer cases and are typically characterised by the abnormal proliferation of malignant white blood cells within the bone marrow (BM) and the presence of tumour cells within the circulation. Specialised compartments, or ‘niches’, within the BM microenvironment play critical roles in housing and maintaining pools of quiescent haematopoietic stem cells (HSCs), and in regulating HSC self-renewal and differentiation [139, 140]. Notably, N-cadherin is expressed by various cell types associated with the HSC niche, including osteoblasts and stromal cells in the endosteal niche, and endothelial cells and pericytes in the perivascular niche [32, 36, 141, 142]. In the following section, we discuss the potential implications of aberrant N-cadherin expression in haematological cancer cells; namely, BM homing and BM microenvironment-mediated protection to chemotherapeutic agents.

### Leukaemia

Leukaemias are thought to arise by the malignant transformation of HSCs into leukaemic stem cells (LSCs) which occupy and modify BM HSC niches [143–146]. Adhesive interactions between LSCs and the BM microenvironment activate signalling cascades which contribute to LSC self-renewal and survival, and the capacity to evade the cytotoxic effects of chemotherapeutic agents [147, 148]. Indeed, therapeutic targeting of adhesion molecules to disrupt interactions with the niche represents a potential strategy to eliminate LSCs [149].

Studies have demonstrated that N-cadherin is expressed in a subpopulation of primitive HSCs [36], but its precise role within the HSC niche in normal haematopoiesis is controversial. To this end, the over-expression of N-cadherin in HSCs has been shown to increase HSC lodgement to BM endosteal surfaces in irradiated mice, enhance HSC self-renewal following serial BM transplantation and promote HSC quiescence in vitro [150]. However, other studies have reported that deletion of N-cadherin in HSCs or osteoblastic cells has no effect on haematopoiesis or HSC quiescence, self-renewal or long-term repopulating activity [141, 151, 152].

While these studies suggest that N-cadherin function may be dispensable in HSC niche maintenance, emerging evidence implicates N-cadherin in the function of the LSC niche. Studies have reported that N-cadherin is expressed on primitive sub-populations of leukaemic cells including patient-derived CD34^+^ CD38^−^ chronic myeloid leukaemia (CML) cells and CD34^+^ CD38^−^ CD123^+^ acute myeloid leukaemia (AML) cells, suggesting that N-cadherin is a marker of LSCs [130, 153, 154]. Similar to solid tumours, N-cadherin is thought to facilitate engagement of leukaemic cancer cells with cells of the surrounding BM microenvironment. For example, treatment of primary human CD34^+^ CML cells with the N-cadherin blocking antibody GC-4 significantly reduced their adhesion to human BM stromal cells (BMSCs) [130]. Similarly, GC-4 treatment of a BCR-ABL-positive mouse acute lymphoblastic leukaemia (ALL) cell line was found to inhibit their ability to adhere to mouse fibroblasts [155]. Pre-clinical mouse models also suggest that N-cadherin may promote BM homing, engraftment and self-renewal of AML cells in vivo [156, 157]. Thus, N-cadherin represents a potential target to inhibit LSC interactions with the BM microenvironment.

#### N-cadherin-mediated cell adhesive interactions promote microenvironmental protection of leukaemic cells to anti-cancer agents

Adhesive interactions between leukaemic cells and BMSCs confer sub-populations of leukaemic cells with resistance to anti-cancer agents, leading to disease relapse [158, 159]. As such, there is growing interest in targeting molecules involved in leukaemic cell-BMSC interactions to enhance leukaemic sensitivity to anti-cancer agents [130, 160]. The role of N-cadherin in the microenvironmental protection of leukaemic cells to anti-cancer agents was first demonstrated in studies showing that N-cadherin expression was associated with resistance to treatment with a farnesyltransferase inhibitor in the murine lymphoblastic leukaemia cell line, B-1, when grown in co-culture with fibroblasts. Enforced N-cadherin expression in B-1 cells also conferred farnesyltransferase inhibitor-resistance when grown in the presence of fibroblasts [155]. Notably, these findings are in line with reports showing that N-cadherin is up-regulated in solid tumour cancer cells resistant to anti-cancer agents [161–164] and androgen deprivation therapy [51, 165]. Direct demonstration that N-cadherin-mediated cell-cell adhesion facilitated microenvironmental protection of leukaemic cells to anti-cancer agents was provided in co-culture experiments with primary human CD34^+^ CML cells and BMSCs. Disruption of CML cell-BMSC adhesion, using an N-cadherin antagonist peptide (containing the HAV sequence) or the N-cadherin function-blocking antibody GC-4 increased CML cell sensitivity to the tyrosine kinase inhibitor imatinib [130, 131]. An association between response to chemotherapy and LSC expression of N-cadherin has also been reported in AML patients. To this end, studies suggest that AML patients exhibiting a higher proportion of N-cadherin-expressing BM-derived CD34^+^ CD38^−^ CD123^+^ LSCs at diagnosis are less responsive to induction chemotherapy [153]. While the precise mechanism by which N-cadherin-mediated adhesion confers drug-resistance in leukaemic cells is unclear, studies in solid tumour cells suggest that N-cadherin-mediated adhesion increases activity of the anti-apoptotic protein Bcl-2, by PI3K/Akt-mediated inactivation of the pro-apoptotic protein Bad [86, 162, 166].

### MM

MM is characterised by the uncontrolled proliferation of transformed immunoglobulin-producing plasma cells (PCs) within the BM. Data from our group, and others, suggest that N-cadherin gene and protein expression is elevated in CD138^+^ BM-derived PCs in approximately 50% of newly-diagnosed MM patients compared with BM PCs from healthy individuals and is associated with poor prognosis [81, 167] (Table 1). Notably, the expression of the N-cadherin gene, *CDH2*, is up-regulated in MM patients harbouring the high-risk t(4;14)(p16;q32) translocation [167, 168]. This translocation encompasses 15–20% of all MM patients and is universally characterised by the dysregulated expression of the oncogenic histone methyltransferase MMSET (also known as NSD2) [169–171]. In addition, *CDH2* expression is also up-regulated in more than 50% of MM patients in the hyperdiploidy-related sub-group [167].

#### N-cadherin promotes MM PC BM homing

The progression of MM disease is underscored by MM PC egress from the primary BM environment and dissemination via the peripheral circulation to distal medullary sites [172]. Functionally, N-cadherin is thought to play a role in MM PC extravasation and homing to the BM. Following intravenous inoculation, the BM-homing capacity of the human MM PC line NCI-H929 in immuno-deficient mice was significantly attenuated by N-cadherin silencing in tumour cells, resulting in increased numbers of residual circulating tumour cells [167]. In addition, N-cadherin knock-down in the murine MM cell line 5TGM1 significantly inhibited adhesion to BM endothelial cell monolayers in vitro, although N-cadherin knock-down or GC-4 antibody-mediated blocking of N-cadherin did not affect the trans-endothelial migration capacity of MM PCs in vitro [167, 173]. Taken together, these data suggest that N-cadherin may promote BM homing of circulating MM PC by facilitating their adhesion to the vasculature, without affecting the rate of subsequent diapedesis.

#### N-cadherin mediates cell-cell adhesion between MM PCs and the BM microenvironment

Adhesive interactions between MM PCs and the BM microenvironment are critical in the permissiveness of the BM to the development of MM disease. These include cell-cell interactions which support MM PC growth and resistance to anti-cancer agents, and promote the inhibition of osteoblast differentiation, thereby contributing to MM PC-mediated bone loss [174, 175]. In addition to endothelial cell adhesion, in vitro studies have demonstrated that N-cadherin mediates the adhesion of human MM PCs to osteoblasts and stromal cells, which constitute the endosteal MM niche [167, 176]. In a functional context, N-cadherin-mediated adhesion between MM PCs and pre-osteoblastic cells has been shown to inhibit osteoblast differentiation, suggesting that N-cadherin may contribute to MM-related bone loss in the clinical setting [167]. Studies have also shown that treatment of human MM PC lines in co-culture with stromal cells or osteoblasts with the N-cadherin blocking antibody GC-4 induced a significant expansion of MM PCs in vitro [176]. Thus, it has been proposed N-cadherin may maintain the proliferative quiescence of MM PC in contact with cells of the endosteal MM niche [176]. In light of the role of N-cadherin in mediating leukaemic cell resistance to anti-cancer agents [130, 131, 155], these findings may provide a rationale to investigate whether N-cadherin-mediated adhesion potentiates resistance to anti-cancer agents in MM.

## N-cadherin as a therapeutic target in cancer

As N-cadherin is widely implicated in cancer metastasis, the utility of N-cadherin antagonists as therapeutic drugs is being investigated in the oncology setting. Notably, N-cadherin-targeting agents have been shown to inhibit cell adhesion and to modulate cell signalling. Interestingly, studies have also shown that N-cadherin-targeting agents affect both tumour cells and tumour-associated vasculature. Here, we describe the current repertoire of N-cadherin antagonists that have displayed efficacy as anti-cancer agents in vivo.

### Monoclonal antibodies

Several monoclonal antibodies directed against N-cadherin have been investigated for their ability to block N-cadherin-dependent tumour migration and invasion in vitro and metastasis in vivo. The mouse monoclonal antibody, designated GC-4, binds to the EC1 domain of N-cadherin monomers and subsequently blocks N-cadherin-mediated adhesion [36, 167, 177, 178]. GC-4 has been shown to suppress N-cadherin-mediated Akt signalling [61, 166], and inhibit the migration and invasion of melanoma, bladder, ovarian and breast cancer cells in vitro [61, 87, 88, 91]. In addition, pre-treatment of AML cells with GC-4 has been shown to inhibit BM homing of circulating tumour cells in vivo [156]. Thus, as N-cadherin plays a role in trans-endothelial migration and BM homing of circulating tumour cells in melanoma and MM, in addition to AML [91, 156, 167, 173], treatment with GC-4 may by therapeutically relevant in the context of limiting the metastatic dissemination of tumour cells in these cancers. Additionally, GC-4-mediated blocking of N-cadherin engagement between human CD34^+^ CML cells and stromal cells increased tumour cell sensitivity to imatinib, demonstrating a potential therapeutic strategy to overcome tyrosine kinase inhibitor resistance [131]. Two additional monoclonal antibodies, 1H7 (targeting N-cadherin EC1–3) and 2A9 (targeting N-cadherin EC4), have shown efficacy in a subcutaneous xenograft prostate cancer mouse model, whereby both antibodies reduced the growth of established tumours and inhibited localised muscle invasion and distant lymph node metastasis [89].

### ADH-1

The lateral clustering of N-cadherin monomers (*cis* adhesion) is essential in the stabilisation and maturation of nascent N-cadherin-mediated adhesive junctions between neighbouring cells [14, 16]. Peptides containing the classical cadherin motif, HAV, are likely to compete with the HAV motif on N-cadherin EC1 for binding to a recognition sequence on EC2 of an adjacent N-cadherin monomer, thereby inhibiting the lateral clustering of N-cadherin monomers [179]. On the basis that a HAV motif located on FGFR-1 is required for FGF-2 binding [112], it is feasible that peptides containing a HAV motif may also inhibit FGFR signalling. This concept led to the development of ADH-1 (N-Ac-CHAVC-NH_2_), a stable cyclic peptide harbouring a HAV motif, which similarly inhibited N-cadherin-dependent function [180]. In vitro, ADH-1 has been shown to induce apoptosis in a range of tumour cell types, and inhibits tumour cell migration at sub-cytotoxic concentrations, with cell sensitivity proportional to relative N-cadherin expression [181–183]. The efficacy of ADH-1 as an anti-cancer agent has been demonstrated in a number of pre-clinical mouse models including pancreatic, breast, colon, ovarian and lung cancer [181, 184]. In addition to inhibiting primary tumour growth, pre-clinical studies also suggest that ADH-1 may inhibit localised tumour invasion and dissemination via the circulation [173, 181]. For example, studies using a mouse model of MM reported that daily ADH-1 treatment commencing immediately prior to, but not after, intravenous inoculation of MM PCs resulted in inhibition of tumour development [173]. Notably, ADH-1 has also been identified as a vascular-disrupting agent, suggesting the compound may have effects on both tumour cells and tumour-associated vasculature [184, 185]. In phase I clinical trials, ADH-1 was shown to have an acceptable toxicity profile with no maximum tolerated dose achieved. ADH-1 treatment was associated with disease control in approximately 25% of patients with advanced chemotherapy-refractory solid tumours, independent of tumour N-cadherin expression status [186, 187].

The therapeutic efficacy of ADH-1 as an anti-cancer agent has been most extensively evaluated in the melanoma setting. Pre-clinical studies suggest that ADH-1 synergistically enhances melanoma tumour response to melphalan [188, 189]. These studies showed that ADH-1 enhances the permeability of tumour vasculature and increases melphalan delivery to the tumour microenvironment, as evidenced by increased formation of melphalan-DNA adducts in tumours. However, the combinatorial effects of ADH-1 and melphalan were not replicated in phase I/II clinical trials [190, 191]. In contrast to other tumour settings, studies have also suggested that ADH-1 may stimulate tumour growth in some mouse models of melanoma [188, 189]. These effects were associated with activation of pro-growth and survival intracellular signalling pathways including Akt signalling and the down-stream mTOR signalling pathway in vitro and in vivo [189]. These data suggest that ADH-1 may act as an N-cadherin agonist in certain tumour contexts. However, to date, ADH-1-mediated activation of tumour cell proliferation and signalling has not been reported in the clinical setting.

## Conclusions

The up-regulation or ‘de novo’ expression of N-cadherin has significant negative implications in metastasis-related cancer relapse and progression, as well as overall survival of cancer patients. In addition to its prognostic significance in cancer, N-cadherin actively promotes the metastatic capacity of tumour cells. Here, we have described three distinct mechanisms by which N-cadherin endows tumour cells with increased migratory capacity: facilitation of collective cell migration, augmentation of FGFR-1 signalling and modulation of canonical Wnt signalling. Unfortunately, our understanding of how N-cadherin influences cancer cell metastasis, and tumorigenesis in general, remains incomplete. Studies in cardiomyocytes, stromal cells and epithelial cancer-like cells have ascribed focal adhesion-like properties to N-cadherin including mechanotransduction and traction-force transmission [192–195]. Indeed, whether a ‘traction and propulsion’-type system, via homotypic N-cadherin mediated cell-cell contacts, is utilised by cancer cells to facilitate migration is intriguing and warrants further investigation. Moreover, there is an emerging body of evidence demonstrating that N-cadherin is expressed and is functionally relevant in the context of numerous haematological malignancies including lymphoblastic and myelogenous leukaemias, and MM. Functionally, pre-clinical studies have demonstrated that N-cadherin promotes the BM homing capacity of circulating MM and leukaemic cells, thereby facilitating metastatic dissemination and intramedullary tumour colonisation [156, 167, 173]. Given N-cadherin is expressed by circulating tumour cells in several epithelial cancers [59, 68, 76] and facilitates trans-endothelial migration in melanoma cells [91, 133], it is tempting to speculate that N-cadherin may also promote tumour cell extravasation in non-haematological malignancies. Studies also suggest that N-cadherin facilitates engagement of LSCs with the tumour microenvironment and promotes leukaemic cell resistance to anti-cancer agents [130, 131, 155]. On the basis of observations in epithelial cancers, N-cadherin may mediate drug resistance in leukaemic cells, at least in part, by activation of the pro-survival protein Bcl-2 [89, 162, 166], or modulation of Sonic Hedgehog signalling [196], widely implicated in cancer stem cell function and maintenance [197]. Interestingly, N-cadherin expression is induced in solid tumour cells resistant to standard anti-cancer agents including tyrosine kinase inhibitors [161–164]. However, it remains to be determined whether N-cadherin functionally contributes to microenvironmental cell adhesion mediated-drug resistance in these cancers.

Given the established role of N-cadherin in cancer, N-cadherin is continually being investigated as a therapeutic target. To date, peptides and mouse monoclonal antibodies have demonstrated some efficacy in the pre-clinical setting, by inhibiting cancer metastasis, enhancing cancer cell sensitivity to chemotherapeutic agents and delaying castration resistance in prostate cancer. However, the challenge remains to develop N-cadherin antagonists which are effective anti-cancer agents in the clinical setting. The humanisation of N-cadherin-blocking antibodies such as GC-4 may represent one such approach to utilise N-cadherin as a therapeutic target. Moreover, the development of next-generation N-cadherin-targeting small molecules with enhanced stability over existing peptide inhibitors show promise as potent inhibitors of N-cadherin function [198–200]. It remains to be seen whether these compounds have efficacy as anti-cancer agents. Undoubtedly, further exploration of N-cadherin as a therapeutic target to inhibit metastasis and overcome drug resistance is warranted.

## Abbreviations

ALL: Acute lymphoblastic leukaemia
AML: Acute myeloid leukaemia
BM: Bone marrow
BMSC: Bone marrow stromal cell
CML: Chronic myeloid leukaemia
EC1: First extracellular domain
EC2: Second extracellular domain
EC4: Fourth extracellular domain
ECM: Extracellular matrix
EMT: Epithelial-to-mesenchymal transition
FGFR: Fibroblast growth factor-receptor
HAV: Histidine-alanine-valine
HSC: Haematopoietic stem cell
LSC: Leukaemic stem cells
MDCK: Transformed canine kidney epithelial cells
MM: Multiple myeloma
PC: Plasma cell
PI3K: Phosphatidylinositide-3 kinase
TCF/LEF: T cell factor/lymphoid enhancer factor
